# Genetics in Keratoconus: where are we?

**DOI:** 10.1186/s40662-016-0047-5

**Published:** 2016-06-27

**Authors:** Yelena Bykhovskaya, Benjamin Margines, Yaron S. Rabinowitz

**Affiliations:** Regenerative Medicine Institute and Department of Surgery, Cedars-Sinai Medical Center, Los Angeles, USA; Cornea Genetic Eye Institute, 50 N. La Cienega Blvd. Suite #340, Beverly Hills, CA 90211 USA; The Jules Stein Eye Institute, University of California Los Angeles, Los Angeles, USA

**Keywords:** Keratoconus, Genetics, Complex disease, Genetic variation, Linkage, Genetic association, Sequencing, Corneal dystrophy, Genotyping

## Abstract

Keratoconus (KC) is a non-inflammatory thinning and protrusion of the cornea in which the cornea assumes a conical shape. Complex etiology of this condition at present remains an enigma. Although environmental factors have been involved in KC pathogenesis, strong underlining genetic susceptibility has been proven. The lack of consistent findings among early genetic studies suggested a heterogeneity and complex nature of the genetic contribution to the development of KC. Recently, genome-wide linkage studies (GWLS) and genome-wide association studies (GWAS) were undertaken. Next-generation sequencing (NGS)-based genomic screens are also currently being carried out. Application of these recently developed comprehensive genetic tools led to a much greater success and increased reproducibility of genetic findings in KC. Involvement of the *LOX* gene identified through GWLS has been confirmed in multiple cohorts of KC patients around the world. KC susceptibility region located at the 2q21.3 chromosomal region near the *RAB3GAP1* gene identified through GWAS was independently replicated. Rare variants in the *ZNF469* gene (mutated in corneal dystrophy Brittle Cornea Syndrome) and in the *TGFBI* gene (mutated in multiple corneal epithelial–stromal TGFBI dystrophies) have been repeatedly identified in familial and sporadic KC patients of different ethnicities. Additional comprehensive strategies using quantitative endophenotypes have been successfully employed to bring further understanding to the genetics of KC. Additional genetic determinants including the *COL5A1* gene have been identified in the GWAS of KC-related trait central corneal thickness. These recent discoveries confirmed the importance of the endophenotype approach for studying complex genetic diseases such as KC and showed that different connective tissue disorders may have the same genetic determinants.

## Background

Keratoconus (KC) is a complex corneal condition characterized by progressive corneal thinning and steepening resulting in moderate to marked visual impairment [1]. The disease is relatively common; it affects approximately 300,000 people in the U.S. [2, 3] and is one of the three top indications for corneal transplantation in the U.S. and worldwide [4].

KC most commonly affects children. It is often detected at puberty and is progressive until the third to fourth decade of life, when it usually arrests [1]. As the cornea thins and steepens, it assumes a conical shape, causing increasingly myopic and astigmatic vision. The decrease in visual acuity can first be addressed with glasses, then later with rigid gas permeable contact lenses. If the disease continues to progress, the contact lens wear becomes gradually more intolerable, and corneal transplantation is indicated to restore vision. These visual changes can also significantly decrease quality of life for KC patients, especially when the patient has been affected for more than a decade, and as the visual acuity of the fellow “better” eye decreases [5].

The progression of the disease is caused by a decrease in the biomechanical strength of the cornea, which is composed primarily of stacked collagen and keratocytes [6]. Current research suggests a complex etiology for the disease including a genetic predisposition [3, 7, 8]. Studies have shown that a positive family history greatly increases the odds of a patient being diagnosed with KC [9–12].

There is also a possible association of KC with other genetic conditions such as inflammatory bowel disease (IBD) [13], Familial Mediterranean Fever (FMF) [14], rare chromosomal abnormalities including those associated with Down syndrome [15], and diabetes mellitus (DM), for which DM patients have a lower incidence of KC [16–18].

However, isolated KC with no associations is by far the most common presentation seen by a practicing clinician [1, 8]. The identification of genes responsible for this type of KC has been the main focus of many studies done by many research groups around the world. Significant progress has been made towards identifying subclinical phenotypic markers suitable for genetic studies by videokeratography and optical coherence tomography, including both anterior and posterior elevation and pachymetric data. As will be shown below, several genes have been implicated across these studies, including genes coding for various collagens and related to extracellular matrix production; still, many others seem to only be tangentially related to these processes. Genetic research into the etiology of the disease will improve the clinician’s ability to predict and ultimately prevent KC in patients.

**Fig. 1:**
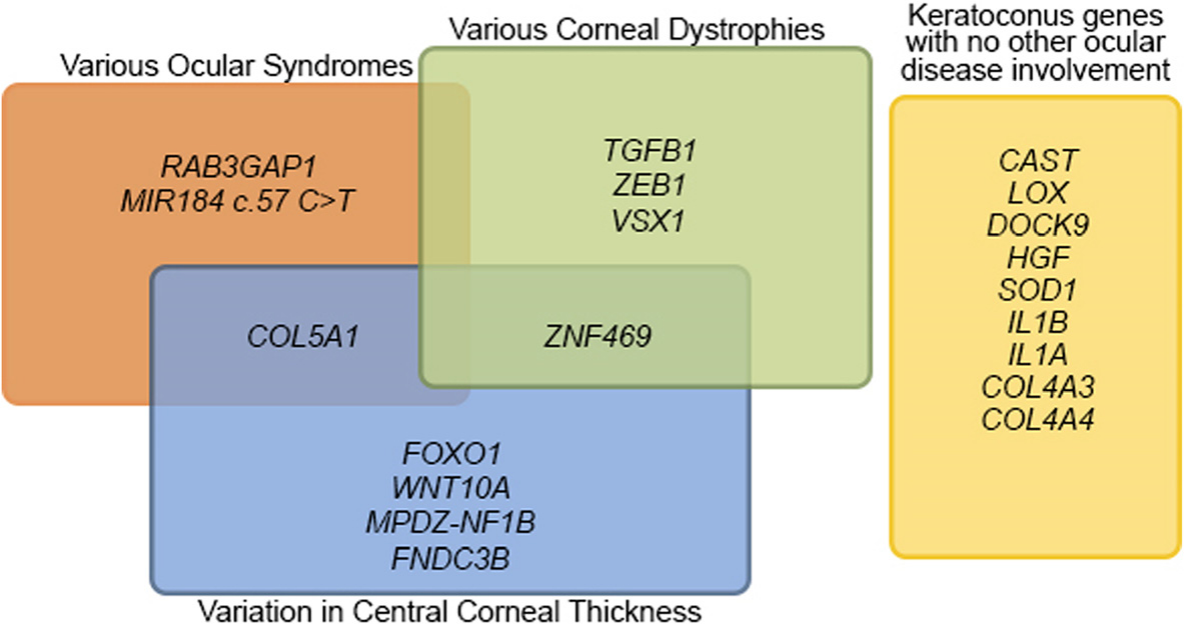
Keratoconus genes and their involvement in other ocular diseases

In the main part below followed by Table 1 and Fig. 1, this paper will summarize the current status of research into the genetics of KC.

**Table 1:** KC genes and identified variants

Gene	Function	CHR	Variant (s)	Method (Reference)	Variant Location	Ref
*LOX*	Lysyl oxidase, participates in collagen cross-linking	5q23.2	rs10519694	GWLS/LD/FM	Intron	[25]
			rs1800449/rs2288393	GWLS/LD/FM	Missense	[25, 29]
				TG		
			rs41407546	S	Missense	UN
			rs2956540	GWLS/LD/FM	Intron	[25, 29–31]
				TG		
*COL5A1*	Collagen type V, alpha-1 chain, part of fibril-forming corneal collagen	9q34.2-q34.3	rs1536482	GWLS/FM/CCT GWAS	5′ near gene	[74, 111]
			rs7044529	GWLS/FM/CCT GWAS	Intron	[74, 108, 111]
*CAST*	Calpain/calpastatin, proteolytic degradation	5q15	rs4434401	GWLS/FM	Intron	[37]
*RAB3GAP1*	Rab GTPase activating protein, regulates exocytosis	2q21.3	rs4954218	GWAS	5′ near gene	[56, 60]
*HGF*	Hepatocyte growth factor, involved in corneal wound healing	7q21.1	rs3735520	GWAS, TG	1 KB promoter	[29, 54]
			rs1014091	GWAS, S	1 KB promoter	[54, 55]
			rs17501108	S	1 KB promoter	[55]
			rs2286194	S	intron	[55]
*FNDC3B*	Fibronectin, extracellular matrix protein	3q26.31	rs4894535	CCT GWAS	Intron	[74]
*FOXO1*	Transcription factor	13q14.1	rs2721051	CCT GWAS, LA CCT GWAS	3′ near gene	[74, 108]
*TGFBI*	Transforming growth factor beta induced	5q31.1	Multiple rare variants	S	Exon	[66, 67]
*ZNF469*	Transcription factor, regulates corneal collagen structure and synthesis	16q24.2	rs9938149	CCT GWAS, TG	3′ near gene	[74, 112]
			Multiple rare variants	S	Exon	[62–64]
*DOCK9*	Dedicator of cytokinesis 9, Guanine nucleotide-exchange factor	13q32.3	c.2262A > C p.Gln754His	GWLS/S	Missense	[41]
*MPDZ-NF1B*	Not available	9p23	rs1324183	CCT GWAS, TG	Intergenic	[30, 74, 112]
*WNT10A*	Member of WNT gene family of secreted signaling proteins	2q35	rs121908120	CCT GWAS	Missense	[113]
*ZEB1*	Zinc finger transcription factor	10p11.22	c.1920G > T; p.Gln640His	S	Missense	[84, 85]
*SOD1*	Superoxide dismutase 1, cytoplasmic antioxidant enzyme	21q22.11	Multiple SNVs, deletion	S	Intron	[99, 115]
*IL1A*	Interleukin 1alpha, cytokine	2q13	rs2071376	TG, S	Intron	[91, 119]
*IL1B*	Interleukin 1beta, cytokine	2q13	rs1143627	TG, S	Promoter	[119–121]
			rs16944	TG, S	Promoter	[119–121]
*COL4A3*	Collagen type IV, alpha-3 chain, structural part of corneal membranes	2q36.3	Multiple SNVs	S	Missense	[123]
*COL4A4*	Collagen type IV, alpha-4 chain, structural part of corneal membranes	2q36.3	Multiple SNVs	S	Missense	[123, 124]
*VSX1*	Visual system homeobox 1, transcription factor	20p11.2	Multiple SNVs	S	Missense, silent, intronic	[133]

*Table abbreviations: KC* = keratoconus; *CHR* = chromosome; *GWLS* = genome-wide linkage study; *GWAS* = genome-wide association study; *LD* = linkage disequilibrium; *FM* = fine mapping; *S* = sequencing; *TG* = targeted genotyping; *CCT* = central corneal thickness; *SNV* = single nucleotide variant; *LA* = Latino; *UN* = unpublished data

## Review

### Genes identified through genome-wide linkage studies (GWLS)

GWLS denotes genotyping families affected by a certain disease using a collection of genetic markers across the genome, and examining how those genetic markers segregate with the disease across multiple families. GWLS, also called linkage studies, were applied successfully to identify genetic variants that contribute to rare disorders like familial breast cancer [19], Huntington disease [20], cystic fibrosis [21], and others (for a comprehensive review see [22]). For decades, these studies were generally conducted using 300–400 microsatellite markers spaced at 10–20 centimorgans (cM) apart. These multiallelic markers were robust and highly informative; however, their genotyping was a time-consuming process. Shortly after single nucleotide polymorphisms (SNPs) were discovered to be abundant polymorphic markers uniformly distributed throughout the human genome [23], dense SNP arrays quickly became the genotyping platform of choice due to the highly unparalleled interrogation and accurate scoring. Testing of genotyping data also evolved from being based on model-based (recessive, dominant, etc.) to robust non-parametric alternatives [24].

### LOX

One of the most significant recent developments in the field of KC genetics is the identification of polymorphisms in the *LOX* (collagen crosslinking enzyme lysyl oxidase) gene that is potentially responsible for a linkage signal at the 5q32-q33 chromosomal region identified by a two-stage GWLS using hundreds of polymorphic microsatellite markers (state-of-the-art-technology available at the time) and the nonparametric method of analysis [25]. After looking at biological functions of hundreds of known or predicted genes in five linkage regions, *LOX* was found to be the most promising candidate among plausible KC candidate genes [26]. *LOX* initiates the cross-linking of collagens and elastin by catalyzing oxidative deamination of the epsilon-amino group in certain lysine and hydroxylysine residues [27]. *LOX* defects can potentially lead to the reduction of cross-linking of collagen fibers of the corneal stroma thus leading to biomechanical weakening of the cornea. Despite the fact that an early study in a group of Italian patients failed to identify *LOX* mutations [28], further extensive genotyping in multiple samples of independently collected KC patients around the world confirmed the effect of SNP rs2956540 in *LOX* in Czech KC cases of European descent [29], Chinese cases [30], Iranian cases [31], and in a recent meta-analysis of published studies [32]. *LOX* involvement is also supported by functional data that showed its attenuation in corneal epithelium of KC patients at levels corresponding to disease severity [33] and revealed changes in *LOX* distribution and its decreased activity in KC corneas [34].

### CAST

Two independent GWLS, one in a single extended KC family and another using multiple unrelated families with KC, mapped a KC locus to a genomic region located at 5q14.3-q21.1 [26, 35]. This region overlaps the *CAST* gene encoding calpastatin, the inhibitor of calpains (non-lysosomal intracellular proteases), which was considered a likely candidate based on the robust presence in the mammalian eye [36], which was further confirmed by *in silico* analysis of EST (expressed sequence tags) databases of human eye tissues [37]. This analysis showed the presence of different *CAST* isoforms in different parts of the eye (cornea, lens, pterygium) as well as a potential difference in their distribution in KC cornea ESTs as compared with those from normal corneal tissues [37]. In addition, higher levels of calpain small subunit-1 protein were found by protein profiling in the epithelia of KC corneas [38]. Initial linkage findings using microsatellite markers were further confirmed by genotyping of high-density SNPs in and around the *CAST* gene in family and case-control panels of patients with KC followed by comprehensive linkage and association analysis [37, 39]. Both studies found *CAST* SNPs to be significantly associated with KC.

### DOCK9

One previously reported chromosomal region at 13q32 linked to KC in Ecuadorian families [40] was sequenced in 51 individuals and 105 matching controls. This mutation screen identified a possible functionally important mutation c.2262A > C (p.Gln754His, rs191047852) in the *DOCK9* gene (dedicator of cytokinesis 9), which segregated with phenotype in one large Ecuadorian family [41]. This particular change was absent in other tested families and in controls, however it is present, albeit with extremely low frequency (minor allele frequency (MAF) = 0.00002; Minor Allele Count = 3) in 61,000 individuals collected around the world and sequenced by The Exome Aggregation Consortium (ExAC). The pathogenic nature of this change is supported by functional investigation, suggesting that it results in the aberrant splicing of the *DOCK9* gene that leads to exon skipping, resulting in the introduction of a premature stop codon, disrupting the functional domains of *DOCK9* protein that may alter the biological role of *DOCK9* as an activator of *Cdc42* (*cell division cycle 42)*, an important regulator of corneal wound repair [42].

### Other loci

Reports on familial KC have proposed both dominant and recessive modes of inheritance, while most families do not fit any typical mode of inheritance [1]. To date, the following gene loci for KC have been identified using GWLS methodology worldwide: 1p36.23-36.21, 2p24, 2q13, 3p14-q13, 5q14.3-q21.1, 5q21.2, 5q32-q33, 8q13.1-q21.11, 9q34, 13q32, 14q11.2, 14q24.3, 15q15.1, 15q22.33-24.2, 16q22.3-q23.1, and 20p13-p12.2, 20q12 [26, 35, 40, 43–51]. Potential mutations in the *IL1RN* (interleukin 1 receptor antagonist) and *SLC4A11* (solute carrier family 4, sodium borate transporter, member 11) genes have been identified in Ecuadorian family linked to the 2q13-q14.3 and 20p13-p12 regions [51].

## Genome-wide association studies (GWAS)

For many years, linkage analysis or GWLS was the primary tool used for the genetic mapping of Mendelian and complex traits with familial aggregation. However, over the last ten years, GWAS have evolved into a powerful tool for investigating the genetic architecture of human genetic diseases, especially complex and common genetic traits. Analytical methods used for GWAS are based on interrogating SNPs, single base-pair changes in the DNA sequence that were found to occur with high frequency in the human genome [23]. SNPs are by far the most abundant and common form of genetic variation in the human genome. Many SNPs are present in a large proportion of human populations [52]. A SNP with allele frequency significantly altered between the case and the control group is considered to be associated with the trait. Minimizing the false positive rate is the most important consideration for GWAS. Thus, genome-wide significance threshold of a nominal p-value < 5 × 10^−8^ has been established [53]. Well-designed GWAS would also include a replication and analyses that include consideration of the joint as well as the individual discovery and replication datasets.

### HGF

Two major GWAS were undertaken almost in parallel and identified new candidate genes for KC. The first one using pooled DNA from an Australian cohort of KC samples and two-step confirmation procedure using two independent case-control cohorts) identified promoter polymorphism SNP rs3735520 in the *HGF* (hepatocyte growth factor) gene [54]. Effect of *HGF* SNP rs3735520 was confirmed in a panel of unrelated Czech KC cases of European descent [29]. Interestingly, extensive analysis of *HGF* SNPs in addition to the Australian KC population identified multiple associated SNPs [55].

### RAB3GAP1

The second GWAS used discovery cohort of 222 Caucasian KC patients and 3324 matched controls and two independent confirmation panels, a case-control panel of 304 cases and 518 controls and a family panel of 307 subjects (146 affected) in 70 families. A novel SNP rs4954218 in the *RAB3GAP1* (RAB3 GTPase activating protein catalytic subunit) gene was identified [56]. Mutations *in RAB3GAP1* gene are associated with the Warburg Micro syndrome in patients of different ethnic backgrounds [57–59]. Notably, identified polymorphism SNP rs4954218 was independently replicated in an unrelated cohort of Australian Caucasian KC patients thus providing further validity to this novel locus [60].

## Genes involved in KC and other corneal dystrophies

### TGFBI

TGF beta-induced protein (TGFBIP) is an extracellular protein that mediates cell adhesion to collagen, laminin and fibronectin and proteoglycans, such as decorin and biglycan with expression changes triggered by the activation of the TGFB signaling pathway [61]. Transcript coding for *TGFBI* (previously called *BIGH3*) was the second most abundant transcript identified in the cDNA library constructed from KC corneas [62]. *TGFBI* gene mutations have been frequently identified in patients with corneal epithelial–stromal *TGFBI* dystrophies, a group of heterogeneous conditions that are characterized by the progressive loss of corneal transparency [63] resulting in the corneal abnormalities witnessed in transgenic mice [64, 65]. Recently, potential mutations in *TGFBI* was identified in Chinese [66] and in Polish KC patients [67]. The *TGFBI* protein has been identified in primary amyloid deposits of hereditary corneal dystrophies and in secondary corneal amyloidosis of diverse etiologies [68] as well as in corneal stromal amyloid deposits in KC patients [69]. Increased levels of *TGFBI* protein have been identified in corneas of patients with Fuchs’ endothelial corneal dystrophy (FECD) [70, 71]. However, not all analyzed KC patients showed association with polymorphisms in the *TGFBI* gene [72].

### ZNF469

Brittle cornea syndrome (BCS) is an autosomal recessive generalized connective tissue disorder associated with extreme corneal thinning (220–450 μm) and a high risk of corneal rupture. Homozygous mutations in the *ZNF469* (zinc finger protein 469) gene coding for a transcriptional factor containing zinc finger domains were found in patients with BCS type 1 [73]. The common genetic variant rs9938149 in *ZNF469* was found to confer increased KC risk [74] and influence CCT (central corneal thickness) in the general population [74–76]. In addition, extensive sequencing of this gene in KC patients of different ethnicities by various research groups identified significant enrichment of a number of potentially pathogenic *ZNF469* alleles [77–79]. These missense variants were found in 23 % of 43 KC patients from New Zealand, one-half of which were Maori or Polynesian [78] and in 12.5 % of three European cohorts with isolated KC (two from the United Kingdom, and one from Switzerland [79]). However, in contrast to previous studies, recent sequencing analysis of *ZNF469* in Polish patients with KC and high myopia and Polish individuals without ocular abnormalities found no significant enrichment of any sequence variants in *ZNF469* [80]. Based on these results together with the lack of evidence for the functional impact of the variants, *ZNF469* involvement remains contentious at this time.

### ZEB1

Mutations in the *ZEB1* (zinc finger E-box binding homeobox 1) gene are repeatedly found in patients with posterior polymorphous corneal dystrophy type 3 (PPCD3) [81, 82] and seem to result in variable ocular phenotypes [83]. In particular, a unique coding mutation c.1920G > T (p.Gln640His) in this gene has been first identified in a family with KC and FECD [84] and later in a patient with triple corneal dystrophy consisting of KC, epithelial basement membrane corneal dystrophy (EBMCD) and FECD [85] thus, further supporting mutational spectrum of *ZEB1* with a unique genotype/phenotype correlation.

### VSX1

The *VSX1* (visual system homeobox 1) gene belongs to a family of homeodomain transcription factors that are thought to control cell differentiation in craniofacial and ocular development, making it a promising functional candidate gene for KC pathogenesis of various corneal dystrophies [86, 87]. Various *VSX1* gene variants have been proposed to be the genetic cause of KC in several sporadic and familiar cases [87], Italian patients [88], Iranian patients [89], Korean [90], and Chinese [91] patients, as well as in cases with PPCD (reviewed in [92]). However, no evidence of association with *VSX1* variants was identified in subsequent recent research studies with large patient cohorts and recently developed genotyping methods that allow for simultaneous interrogation of hundreds of thousands of independent SNPs providing information on common genomic variation [93–99]. In addition, extending previously identified *VSX1* variants to additional populations and samples provided evidence of their benign nature [100, 101]. Current evidence seems to largely support a limited role for *VSX1* in KC pathogenesis.

## Use of endophenotype CCT to identify additional genes and variants

Variation in CCT is one of the most highly heritable human traits [102, 103]. Reduced CCT is often associated with KC [104, 105]. Several recently performed GWAS identified a number of genomic loci associated with differences in CCT that revealed differences in genetic determinants of CCT between ethnic groups, and evaluated the relevance of CCT-associated loci to KC susceptibility [74–76, 106–108].

### COL5A1

Since the corneal stroma is composed of collagen fibrils, it is not surprising that a number of genomic loci associated with CCT contain genes that code for various collagens, such as *COL1A1* and *COL1A2* [109], and *COL8A2* [76]. The most evidence, however, points to the involvement of the *COL5A1* gene coding for type V collagen subunit 1 in both CCT variation [76, 106, 108, 110] and KC [74, 111]. However, in-depth analysis of variants in KC families as well as in sporadic cases showed that while some KC patients carrying minor alleles of these variants do have thinner corneas, others do not, highlighting the complex relationship between genetic variation in *COL5A1*, corneal thinning and KC development [111].

### FNDC3B, FOXO1, MPDZ-NF1B

CCT-associated variants rs4894535 located in the *FNDC3B* gene, rs2721051 near the *FOXO1* gene, and rs1324183 located between the *MPDZ* and *NF1B* genes have been found to be associated with KC in large multinational cohorts of KC patients and controls [74]. SNP rs1324183 was further associated with an increased risk of KC in Chinese cases [30] and in the Australian population [112].

### WNT10A

The identified CCT variants only explain about 8 % of the variability of the trait [75]. One possible component of the missing heritability is low frequency variants. The published GWAS of CCT to date focus primarily on common variants (i.e., MAF >5 %); however, when putative rare functional coding exome variants from the Illumina Human Exome array were evaluated, a novel rare *WNT10A* exonic variant (rs121908120), which increases the risk of KC by decreasing corneal thickness, was identified [113]. This variant is located in a gene 437 kb away from the *USP37* gene, previously associated with CCT, and completely accounts for the signal previously seen at *USP37*. It increases the risk of KC two times. *WNT10A* (wingless-type MMTV integration site family member 10A) belongs to the *WNT* gene family. This family consists of structurally related genes encoding secreted signaling molecules that have been implicated in important developmental processes, including regulation of cell fate and patterning during corneal development [114].

## Other potentially involved genes

### SOD1

The *SOD1* (superoxide dismutase 1) gene has been proposed and repeatedly investigated as a candidate gene for KC with published data supporting [99, 115] as well as refuting [28, 40, 89, 93, 116] its involvement. The sometimes identified increased levels of oxidative stress markers in corneas from patients with KC [117, 118] suggest that defects in the *SOD1* gene, encoding a major cytoplasmic antioxidant enzyme that metabolizes superoxide radicals, might be involved in the development of this disease. However, lack of data supporting such genetic involvement suggests a possibility that said oxidative stress may be an end product of other pathologic processes caused by defects in other genes.

### IL1B, IL1A

*IL1B* (interleukin 1 beta) promoter polymorphisms and *IL1A* (interleukin 1 alpha) intronic polymorphism rs2071376 have been suggested to play roles in KC susceptibility due to significant differences in allelic frequency between groups of KC patients and controls in Han Chinese [91, 119], Korean [120], and Japanese [121] populations. However, the same polymorphism showed no evidence of association in the Turkish population [122].

### COL4A3, COL4A4

Genes coding for collagens *COL4A3* (type IV collagen alpha3, *COL4A4* (type IV collagen alpha4) have been suggested as well [123, 124]; however, additional studies in multiple populations found no association with these SNPs [91, 125] or found evidence of their extensive presence in the normal population [126].

## Rare recurring mutation in *miR184* (microRNA184) in families with KC and cataracts

MicroRNAs (miRNAs) bind to complementary sequences within the 3′ untranslated region (UTR) of mRNAs from hundreds of target genes, leading either to mRNA degradation or suppression of translation. Germline sequence variants in mature miRNAs are extremely rare possibly due to the extreme conservation and importance of mature miRNAs as well as its tremendously small size (18–25 base pairs). A heterozygous c.57 C > T mutation in the seed region of *MIR184* (miR-184) was found to be responsible for familial severe KC combined with early-onset anterior polar cataract in the Northern Irish family [127]. The same mutation was later identified in an unrelated family with the EDICT (endothelial dystrophy, iris hypoplasia, congenital cataract, and stromal thinning) syndrome [128] and most recently in a five-generation family with cataracts and varying corneal abnormalities including severe KC and non-ectatic corneal thinning from Galicia, Spain [129]. Interestingly, genetic ancestry testing of the Spanish family strongly suggested that the c.57 C > T *MIR184* mutation arose independently in the Galician and Northern Irish families and thus represents the first observation of the recurrent germline mutation in the microRNA gene leading to the genetic disease described [130]. No mutation(s) within the stem loop of *MIR184* in isolated KC cases was detected in two independent screens, suggesting that mutations in *MIR184* are more relevant to cases of KC associated with other ocular abnormalities [131, 132].

## Discussion

Major strides had been made in the understanding of KC genetics. However, more research is needed to make biological connections between already identified KC genes as well as new genes. Several KC research groups around the world are designing and performing high-throughput studies in familial and sporadic KC patients to accomplish this task. These studies will include genotyping large cohorts of well-characterized ethnically homogenous patients and large groups of ethnically matched controls using the most comprehensive genomic chips containing up to 2.5 million independent SNPs. In addition, outgoing development and dropping prices for the next-generation sequencing (NGS) based whole genome screens are becoming more of a reality. Such screens especially in families with KC and in thoroughly selected KC patients with extreme phenotypic features can identify and test rare or low frequency variants that cannot be tested with chip technology.

As KC often begins by affecting vision, and thereby quality of life at a young age, being able to diagnose and arrest the progress of the disease at an earlier stage will aid clinicians in treating KC patients. Currently, KC is diagnosed by evaluating a variety of non-genetic metrics, such as corneal topography and pachymetry. A thorough understanding of the genetic contribution to the disease progression will increase the certainty of a KC diagnosis and allow that diagnosis to be made sooner. It may also bring additional options for the treatment of this disorder. As technology progresses and genetic screening becomes simpler and more cost effective, ophthalmologists may find value in testing suspected KC patients for the genetic variations discussed in this paper. This knowledge could enable the general ophthalmologist to understand the disease etiology such that he or she can diagnose more easily and potentially screen patients’ family members, thereby proactively caring for the disease as it develops.

## Conclusion

Although the genetic etiology of KC remains to be comprehensively defined, recent GWLS and GWAS have made significant progress in identifying genetic variation that is strongly correlated with the disease. SNPs associated with the following genes have been implicated: *LOX, CAST, DOCK9, IL1RN, SLC4A11, HGF, RAB3GAP1, TGFBI, ZNF469, ZEB1, VSX1, COL5A1, COL4A3, COL4A4, FNDC3B, FOXO1, MPDZ-NF1B, WNT10A, SOD1, IL1B, IL1A*, in addition to the microRNA *MIR184*. Notably, not all analyses of each of these genes completely confirm their role in KC pathogenesis. Rather, it is likely that KC can result from abnormalities in several biochemical pathways for which the interactions have not yet been outlined. Genetic analyses that document these associations will eventually elucidate this connection.

## Abbreviations

CCT, central corneal thickness; cM, centimorgan; GWAS, Genome-wide association study; GWLS, Genome-wide linkage study; KC, Keratoconus; LD, Linkage disequilibrium; NGS, next-generation sequencing
